# Assessing the quality of deliberative stakeholder consultations involving allied health professionals in pediatric palliative care and hematology/oncology in Canada

**DOI:** 10.1186/s12904-021-00884-2

**Published:** 2021-12-15

**Authors:** Vasiliki Rahimzadeh, Cristina Longo, Justin Gagnon, Conrad Fernandez, Gillian Bartlett

**Affiliations:** 1 Stanford Center for Biomedical Ethics, 300 Pasteur Drive, Stanford, CA 94305 USA; 2 Faculty of Pharmacy, Université de Montréal, Montréal, QC, Canada; 3 Department of Family Medicine, McGill University, Montréal, QC, Canada; 4 Departments of Pediatrics and Bioethics Head, Division of Pediatric Hematology/Oncology Dalhousie University and IWK Health Centre, Halifax, NS, Canada; 5 Department of Family and Community Medicine, University of Missouri School of Medicine, Columbia, MO USA

**Keywords:** deliberative democracy, quality assessment, stakeholder consultation, pediatric oncology, palliative care

## Abstract

**Background:**

In this paper we assess the quality of six deliberative stakeholder consultations regarding the implementation of a precision diagnostic for life-threatening pediatric brain tumors. Decision makers who base policy recommendations on the outputs of consultative exercises can presuppose that all deliberants are well informed of the policy issue, that participation in the deliberative process was fair, and that overcoming implementation barriers will necessarily result in practice change. Additional evidence is therefore needed to substantiate the informational quality of the deliberation, measure the equality of participation and study the effects on stakeholder reasoning to appropriately guide uptake of proposed recommendation(s).

**Methods:**

Using the DeVries framework for assessing the deliberative quality, we analyzed data from 44 post-consultation evaluation surveys completed by pediatric oncology and palliative care teams at two tertiary pediatric healthcare centers in Canada. We also conducted turn-taking and word-contribution analyses from the text transcriptions of each deliberation to assess *equality of participation* using descriptive statistics.

**Results:**

Deliberants agreed the quality of the deliberative *process* was fair (median ratings ranging from 9–10 out of 10) and the opportunities to receive expert information and discuss with others about the implementation of a new LDT were helpful (9.5 out of 10). While the session improved understanding of the implementation barriers and opportunities, it had marginal effects on deliberants’ reasoning about whether LDTs would change their own clinical practice (3–10 out of 10). Participation was proportionate in at least four of the six deliberations, where no deliberant took more than 20% of total turns and contributed equal to, or less than 20% of total words.

**Conclusion:**

The quality assessment we performed demonstrates high informational value and perceived fairness of two deliberative stakeholder consultations involving pediatric palliative care and oncology teams in Canada. Quality assessments can reveal how the process of deliberation unfolds, whether deliberative outputs are the result of equitable participation among deliberants and what, if any, stakeholder voices may be missing. Such assessments should be routinely reported as a condition of methodological rigor and trustworthiness of deliberative stakeholder engagement research.

**Supplementary Information:**

The online version contains supplementary material available at 10.1186/s12904-021-00884-2.

## Background

Brain tumors are the leading cause of cancer related death in children and malignant gliomas contribute an important component to this number [1]. Advanced understanding of the basic biology and pharmacogenomics of pediatric brain tumors portend a clinical future in which oncologists base their clinical decisions in large part on results from laboratory derived tests (LDT) using next generation sequencing [2]. In a seminal 2017 paper, researchers characterized 85 different mutations associated with diffuse midline gliomas, an especially aggressive brain tumor in children. This discovery prompted development of a laboratory derived test (LDT) that could molecularly diagnose tumor types, stratify patients according to risk and guide treatment decisions. Of these, H3 K27 mutations are resistant to all known therapies, and detection using the LDT carries a universally fatal prognosis. Palliative care is thus currently standard therapy for the ~20% of glioma patients with a confirmed K27M variant.

Despite advances in LDT development for pediatric gliomas, sparse psychosocial research has investigated the ethical and social impacts of introducing an LDT for diagnosing K27 mutations in the clinic in standard practice, nor how to best support communication with patients and their families in light of a potentially catastrophic diagnosis the LDT may indicates [3–7].

We previously conducted six deliberative stakeholder consultations with healthcare professionals [3, 8] across two large, pediatric academic medical centers in Canada to fill this knowledge gap. Deliberative stakeholder consultations draw on deliberative democratic theory and rest on two interrelated ideas: that stakeholder communities, both lay and expert have experiential knowledge, and that such knowledge should be reflected in making decisions about whether, and how new policies are enacted [9, 10]. The consultations unveil normative values specific to a forthcoming policy or practice change from the perspectives of key stakeholders likely to enact the policy or change, and allow stakeholders to make recommendations for implementation [11–13]. Deliberants freely debate, for example, the ethical, social and clinical implications of policies and in the process generate both deliberative^1^ and analytical^2^ outputs [14].

Whereas myriad survey methods can be applied to assess whether and to what degree understanding and behavior changes [15], “if the deliberative process is to be trusted, we need to know more about what happens as people deliberate*”* [16]*.* Without quality assessment of deliberative exercises, policy recommendations could disproportionately reflect the perspectives and interests of dominant actors; reinforce power imbalances and hierarchies; as well as mask values from stakeholders who may be more vulnerable, but no less essential to the policy development process. It is worth noting that while discourse analysis and democratic praxis are key sociological phenomena relevant to deliberative democratic engagement, no study to our knowledge has yet systematically compared deliberative outcomes with and without quality assessment of the deliberative process.

Previous studies have captured deliberative quality by probing the effects of deliberation on participants’ content knowledge, including whether deliberants changed their opinions, the extent to which deliberants felt their recommendations heeded new information as well as how deliberants perceived the overall value of deliberative experience [see for example 18]. DeVries et al. propose a practical framework oriented around assessing the following indicators for deliberative quality: synthesized key factors that influence deliberative quality into a practical framework for quality assessment: *process*, *information* and *reasoning* [17]. P*rocess* refers to how deliberation is designed and the implementation of those design elements throughout the deliberative exercise. It is measured by assessing the degree of facilitation, equality of participation, participant engagement and self-reported feelings of mutual respect among deliberants. *Information* aims to assess how deliberants respond to new information and the effects of this information on deliberant perspectives. Metrics to assess *information* include how effectively content experts relay accurate information, how well deliberants understand and incorporate this information into their discussions, and how (if at all) the deliberation influenced opinions. Lastly, *reasoning*, aims to measure how effectively a group considers alternative viewpoints in their efforts to reach collective agreement on a policy position.

In this paper, we report findings from a deliberative quality assessment of six stakeholder consultations on the basis of *process* and *information* using criteria adapted from the validated survey instrument developed by DeVries et al. [17]. Our assessment was guided by the following question: What was the deliberative quality of six stakeholder consultations held with healthcare professionals working in pediatric oncology and palliative care at tertiary care centers in Canada? Unlike for *process* and *information*, quality metrics for *reasoning* can be context- and population specific because they rely on how deliberants justify their arguments, the diversity of ethical, legal and social norms guiding practice, and how amenable existing practices are to alternative policy options. For these reasons we modified the approach outlined in the original DeVries et al. framework for assessing *reasoning. We instead* conducted ethnographic observations which provided richer accounts of the clinical decision-making contexts unique to our policy topic of interest (i.e. implementation of LDT). We elaborate on our methodological rationales underpinning this revised approach elsewhere [8].

### Participants

Because deliberative stakeholder consultations aim to assess implementation in a specific practice context [18], the clinical teams recruited to participate in our study were unique to those ‘mini-publics’ that interact with glioma patients and their families [19]. We purposively recruited 48 clinical staff at two tertiary medical centers in Canada. Pediatric glioma patients in the region are referred to these centers for specialty care. At the time of the deliberations, researchers planned to launch a pilot program of the LDT to diagnose the K27 mutation and subsequently assign care options based on these results. We obtained survey data from oncology teams (*N* = 18), palliative care teams (*N* = 7) and from mixed palliative care and oncology teams (*N* = 23) across both healthcare centers. Clinical teams comprised of physicians, nurses, social workers, medical students, and fellows.

## Methods

We administered a post-consultation evaluation survey developed by DeVries and colleagues [17] to assess the quality of *information* and *process* to 48 deliberants between March 2015 and August 2016 (See Additional File 1). Deliberants rated their deliberative experience and self-reported learning on a 10-point Likert scale. Deliberants had limited knowledge about how the LDT was developed, its sensitivity, specificity, or clinical validity and utility, and little to no clinical experience with ordering the LDT as it was not yet a standardized tool for diagnosing the K27 variant. An expert facilitator gave an informational presentation at the consultation start, which covered background evidence on the current i) incidence, prevalence, and burden of disease of pediatric high-grade glioma; ii) mortality data for specific high-grade gliomas variants; and iii) sensitivity and specificity of a newly developed LDT to detect high grade glioma variants. Deliberants were invited to ask the facilitator any clarifying questions in response to the information presented and an open discussion followed. Deliberants discussed what information would be needed to effectively communicate LDT results to patients and families, whether communication needs varied when the LDT indicated a lethal mutation, and to what extent the LDT would driv of treatment planning or significantly affect clinical practice.

The deliberative stakeholder consultations were shortened compared to those described in the literature [20] to accommodate for the extreme time constraints of healthcare professionals, and to better account for the rigid professional hierarchy in medicine e.g. between attendings, residents and medical students. The interprofessional dyanmics between professionals in subacute medical specialties like pediatric hematology/oncology and palliative care motivated us to conduct a separate assessment of *participation equality*, a subcategory of deliberative *process* [13]. We first transcribed all deliberations verbatim, and then conducted turn-taking analyses on the text transcriptions from each deliberation. We counted a turn anytime there was a change in speaker, and totaled the words spoken during their turn. We then calculated the i) percent participation of deliberants across the six deliberations, tallied the ii) total number of turns taken and counted iii) total words spoken. We considered a deliberative session to have achieved proportionate participation when no one individual took more than 40% of total turns or spoke more than 40% of all words.

### Analysis

Descriptive statistics, including group mean, median and range were calculated from survey ratings. We compared group means across all six deliberative stakeholder consultations to determine how deliberants perceived the quality of the information they received and fairness of the deliberative process. We established two thresholds, more than 20% and more than 40% to capture unequal participation in turn taking or words spoken, respectively, based on the total N for each deliberative event.

## Results

### Process

Three survey questions probed deliberants’ perspectives on the deliberative *process* (Table 1). Median ratings for feeling that one’s opinions were respected by the group ranged from 9.5–10 across all deliberation groups. Ratings were similarly high in response to whether deliberants felt listened to and whether the process was fair, both ranging from 9–10. Relative to palliative care and mixed teams, oncology teams reported the lowest average and median ratings on each of the four process-related prompts. Oncology-only and mixed teams at Hospitals 1, and oncology-only teams at Hospital 2 were also less likely on average to abide by the group’s final position to implement the LDT (8.1, 8.35 and 8.7, respectively).

**Table 1:** Mean and median scores calculated from 48 post-consultation evaluation surveys from six separate consultations using the De Vries et al. framework [17] to assess quality of deliberative *process* and *information*. Deliberants rated survey items on a 10-point Likert scale (1 being not at all, 10 being very much)

		Hospital 1			Hospital 2		
	Survey question	Oncology, ***N*** = 8	Palliative Care, ***N*** = 3	Mixed, ***N*** = 14	Oncology, ***N*** = 10	Palliative Care, ***N*** = 4	Mixed, ***N*** = 9
		Mean (Median)	Mean (Median)	Mean (Median)	Mean (Median)	Mean (Median)	Mean (Median)
*Process*	Do you feel that your opinions were respected by your group?	9.13 (9.5)	9.66 (10)	9.42 (10)	9.4 (10)	10 (10)	10 (10)
	Do you feel you were listened to by your facilitator?	8.88 (9.5)	10 (10)	9.5 (10)	9.6 (10)	10 (10)	10 (10)
	Do you feel that the process that led to your group’s response was fair?	9 (9)	10 (10)	9.6 (10)	9.3 (9.5)	10 (10)	10 (10)
	How willing are you to abide by the group’s final position, even if you personally have a different view?	8.13 (9)	9.66 (10)	8.35 (9)	8.7 (9)	9.5 (9.5)	9.1 (10)
*Information*	How helpful did you find question and answer interaction with the experts?	8.13 (9)	9.66 (10)	8.6 (9)	8.3 (9)	9.5 (9.5)	9.1 (9.5)
	How helpful did you find the formal presentations given by the experts?	6.63 (8)	9.66 (10)	7.8 (9)	8.6 (9)	10 (10)	8.8 (9.5)
	How helpful did you find discussing the issues with other participants?	7.5 (9)	10 (10)	8.8 (9)	8.6 (10)	10 (10)	9.6 (10)
	How much did attending the session change your understanding about the use of this new pharmacogenomics test in pediatric oncology?	5.75 (6)	9.33 (10)	8.1 (9)	4.5 (5)	1.75 (2)	7.2 (7)
	How much did attending the session change your opinion about the use of this new pharmacogenomics test in pediatric oncology?	4.5 (5)	8.33 (10)	6.8 (7)	4.3 (5)	3 (3)	7.1 (8)

### Equality of participation

Table 2 summarizes the results of the turn-taking and word count analyses. Four of six deliberations achieved 100% deliberant participation where every deliberant contributed to the discussion at least once. We analyzed the highest number of turns taken among deliberants in the mixed group at Hospital 2 and the lowest among deliberants in the oncology groups also at Hospital 2. Despite similar participation numbers, the palliative care group at Hospital 1 took nearly twice the number of the turns than the palliative care group at Hospital 2. Although the latter reported the least number of turns, it registered the highest number of total words spoken of any group assessed in our study. The palliative care group at Hospital 2 was moreover the only group where one deliberant contributed more than 40% of the total words.

**Table 2:** Summary of turn-taking analysis for six deliberative stakeholder consultations

	Hospital 1			Hospital 2		
	Oncology (***N*** = 8)	Palliative Care (***N*** = 3)	Mixed (***N*** = 14)	Oncology (***N*** = 10)	Palliative Care (***N*** = 4)	Mixed (***N*** = 9)
**% participation**	87.5	100	85	100	100	100
**Total number of turns taken**	193	207	125	87	108	214
**Total number of words spoken**	6700	8419	5541	6166	10,448	9110
**Number of deliberants taking more than**						
20% of total turns	2	2	1	2	2	1
40% of total turns	0	0	0	1	0	0
**Number of deliberants speaking more than**						
20% of total words	1	1	1	3	1	1
40% of total words	0	0	0	0	1	0

### Information

Deliberants generally perceived the informational content presented during the presentation to be helpful, and that discussions with both the expert as well as with other discussants facilitated their understanding of the LDT and its proposed implementation. Palliative care teams reported higher median ratings for each of the first three *information*-related prompts referring to the informational presentation, interaction with the expert and group discussion (from 9–10 out of 10). Medians from oncology only teams were slightly lower on each of these items in comparison (8–10).

With the exception of the palliative care-only team at Hospital 1, ratings in response to the question *How much did attending the session change your understanding about the use of this new pharmacogenomics test in pediatric oncology*? and *How much did attending the session change your opinion about the use of this new pharmacogenomics test in pediatric oncology?* were the lowest in the separated professional sessions at Hospital 2. We observed a measurable increase in ratings among deliberants in the mixed group at Hospital 2—nearly 5 Likert points higher in some cases—when compared to deliberations with oncology or palliative care teams alone. We observed a similar pattern between the mixed group and the oncology group at Hospital 1. The palliative care only team at Hospital 2 reported the lowest median change in understanding (2 out of 10) and opinion (3 out of 10) concerning LDT use of any surveyed group in the study.

## Discussion

Deliberants generally agreed the quality of the deliberative *process* was both fair and respectful of the groups’ diverse opinions. The early stage of LDT research and development meant that most clinicians were unlikely to have taken part in prior discussions about implementation. Presenting information in a comprehensive, yet accessible way was therefore critical to facilitating an informed discussion among deliberants and substantiates *information* as a necessary metric for deliberative quality [15]. The shorter deliberative sessions to accommodate the intense time constraints of practicing clinicians in our study sample further reinforce the importance of conducting this deliberative quality assessment [12].

Based on average ratings for the survey items that assessed *information* quality, attending the session had moderate effects on deliberants’ overall understanding of the new LDT, and little to no effect on anticipated change to future clinical practice. Ratings among the palliative care group at Hospital 2 demonstrated the starkest finding in this regard. Despite unanimous agreement that the presentation, expert question and answer session and peer discussion were adequately informative (median 9.5–10)—all survey items probing *information* quality—the median rating of a deliberant’s self-reported change in understanding and opinion of the LDT was 1.75 and 3, respectively. One possible explanation for why palliative care teams were unmoved by the information is that, ultimately, the responsibility for conveying curative treatment options rests with the oncologist.

Our finding is consistent with a randomized control trial comparing four different deliberative methods in healthcare/health policy decision making. Brief citizen deliberations, most akin to the stakeholder consultation model adopted in this study, resulted in no statistically significant change in deliberants’ preferences on healthcare decisions. The authors of the study further noted “one may be more willing to accept the importance of knowing about medical evidence … before one would accept that evidence is more important than preferences. Deliberation had an impact on the “lower bar” of perceived importance but stopped short of the strongest changes in attitude in this domain” [21].

Deliberations within the mixed group  rated *information* quality higher than palliative care-and oncology-only groups at Hospital 2, but lower at Hospital 1. Several hypotheses may help to explain this finding. It is well documented in the literature that palliative care teams are more often consulted as a child’s condition deteriorates and where such services are available. They are seldom included as part of the interdisciplinary oncology team at diagnosis or early in the patient’s care journey [22–24] when research demonstrates their involvement can greatly improve pain and symptom management and health-related quality of life, among other possible clinical benefits [25]. It is possible that low ratings in response to whether the sessions changed professional understanding or opinion may be relative to how deliberants perceive the integration (or lack thereof) between palliative care and oncology teams at the diagnostic stage in which the LDT will be implemented. This finding corroborates the patient decision-making and communication literature in the care of critically ill children with cancer [26].

There is some debate in the literature on the extent to which unequal participation lessens the quality of deliberative outputs [27]. Our *equality of participation* assessment demonstrated that turn-taking was not discipline-specific nor correlated with group size. We observed unequal participation—more than 40% of total turns taken and words spoken by one deliberant—during two deliberations (Table 2). While it does not invalidate the perspectives or the deliberative outputs [14], this threat to deliberative quality should be taken into account when using study findings to guide practice recommendations. Disproportionate participation at Hospital 1 did not, however, adversely affect perceptions of procedural fairness or respect as evidenced by the high ratings reported on relevant survey items. Democratic deliberation is a transformative research process intended to draw out opinion, identify and potentially resolve points of conflict, and inform action. For the practical purposes of initiating discussion and informing action, discussion need not necessarily be proportionally equivalent.

### Limitations

Our findings should be considered in light of several limitations. First, we targeted recruitment of healthcare professionals at the few tertiary academic health centers where the newly developed LDT would be piloted. Albeit a small sample size, this recruitment approach achieved the specificity desired to identify implementation challenges among those on the frontlines of implementation in the near future. It is therefore also possible that our responses may reflect a social desirability bias as a result. The deliberative stakeholder consultation itself is vulnerable to several methodological critiques, namely that findings can disproportionately reflect the viewpoints and perspectives of the most dominant voices. While an imperfect proxy, our quantitative measures of *process* and *information* using a validated survey instrument*,* and *equality of participation* by analyzing turn-taking and deliberative quality, helped to determine the nature and extent of any power imbalances in the consultations. Qualitative interviews could, in addition to evaluation surveys, provide richer qualitative evidence for assessment, particularly deliberative *process* and *equality of participation* criteria. Lastly, deliberative forums on highly sensitive topics have been criticized for their potential to engender further polarization. Our decision to first conduct separate, followed by mixed deliberations with oncology and palliative care teams attempted to reduce this polarization, but may have contributed to underestimates in both *information* and *process* quality. Indeed, dominance was a concern for the two discipline-specific deliberation groups at Hospital 1 when compared to either of the mixed groups.

## Conclusion

This study assessed the deliberative quality of six stakeholder consultations with healthcare professionals in pediatric oncology and palliative care across two tertiary care centers in Canada. The cumulative results demonstrated that deliberants agreed the i) overall quality of the deliberative *process* was fair, ii) the opportunity to receive quality *information* about LDT implementation and discuss the challenges and opportunities with an expert was helpful, and that iii) *participation* was equal among deliberants in four deliberations we held. We found that *equality of participation* was challenged in two deliberations. One deliberant took more than 40% of turns, and another spoke more than 40% of total words. These imbalances did not, however, appear to negatively affect deliberants’ perception of fairness or respect during the session according to survey results. Assessing deliberative quality not only enhances the rigor and trustworthiness of substantive outputs from deliberative exercises, but it also enables empirical study of how differences in quality may influence consensus building processes. More contributions to the methods literature are needed to investigate these differences systematically before making such assessment part of standard research practice.

## Supplementary Information

**Additional file 1.** Evaluation of democratic deliberation session. An English language copy of the post-deliberation questionnaire adapted from De Vries and colleagues (2011) is provided in Additional File 1.

## Data Availability

The datasets used and/or analyzed during the current study are available from the corresponding author on reasonable request.

## Abbreviations

**LDT**: Laboratory derived test

## Footnotes

1 Deliberative outputs are syntheses of collective decisions, opinions and statements from deliberants on the policy issue at hand, with limited attention to the discursive elements underpinning them.

2 Analytical outputs, in contrast, are those which “treats participants’ statements as socio-cultural products, contingent on the discursive context within which they are produced” [14].
