# Supplementary material for: Assessing the quality of deliberative stakeholder consultations involving allied health professionals in pediatric palliative care and hematology/oncology in Canada
Source: BMC Palliat Care. 2021 Dec 15;20:189. doi: 10.1186/s12904-021-00884-2 (PMC8672505; doi:10.1186/s12904-021-00884-2)
Supplement: Supplementary file 1 — Additional file 1. Evaluation of democratic deliberation session. An English language copy of the post-deliberation questionnaire adapted from De Vries and colleagues (2011) is provided in Additional File 1. [file 12904_2021_884_MOESM1_ESM.docx]

**Additional file 1.** Evaluation of the Democratic Deliberative Session^[[1]](#footnote-1)^

Thank you for participating in this deliberative stakeholder consultation. Please answer the following questions using a 10 point scale, where 1= Not at all,

10 = Very Much.

1. Do you feel that your opinions were respected by your group?


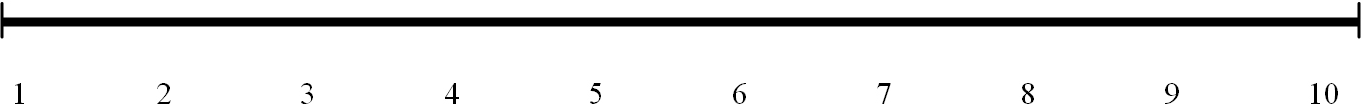


1. Do you feel you were listened to by your facilitator?


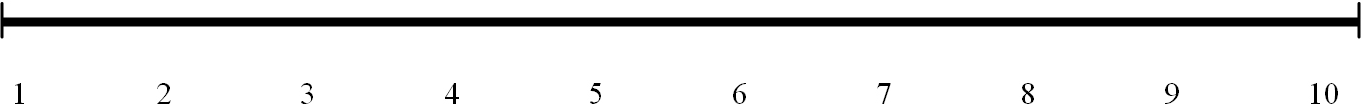


1. Do you feel that the process that led to your group’s response was fair?


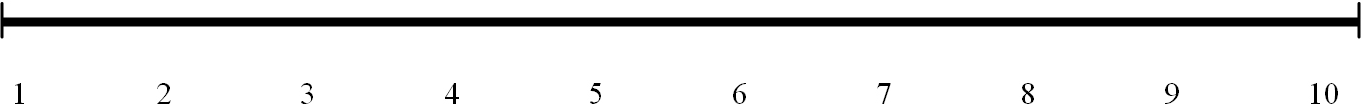


1. How willing are you to abide by the group’s final position, even if you personally have a different view?


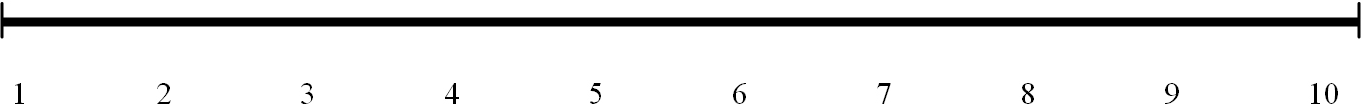


1. How helpful did you find each of the following?
   1. Question and answer interaction with the experts?


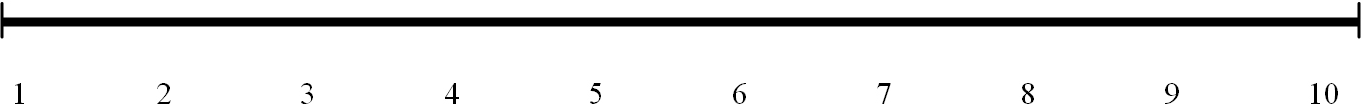


- 1. The formal presentations given by the experts?


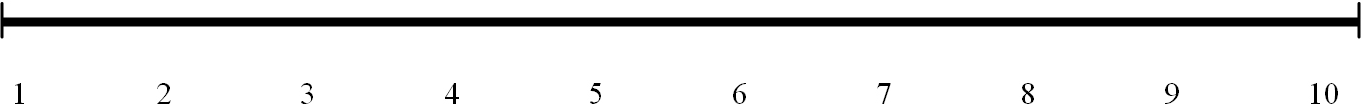


- 1. Discussing the issues with other participants?


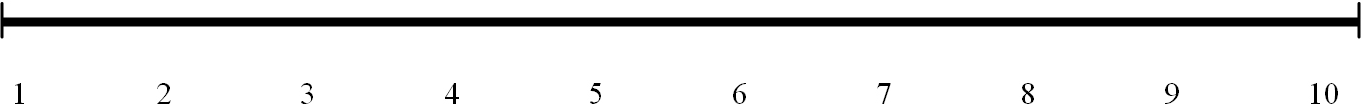


1. How much did attending the session change your *understanding* about the use of this new pharmacogenomic test in pediatric oncology?


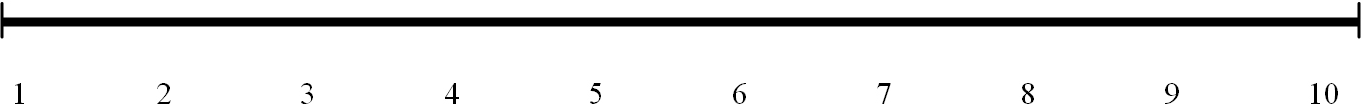


1. How much did attending the session change your *opinion* about the use of this new pharamcogenomic test in pediatric oncology?


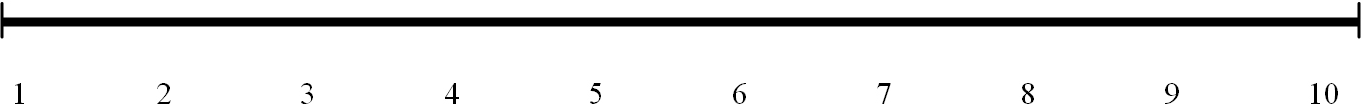


1. Adapted from DeVries and colleagues (2011) [↑](#footnote-ref-1)
